# Alpha-santalol, a chemopreventive agent against skin cancer, causes G_2_/M cell cycle arrest in both p53-mutated human epidermoid carcinoma A431 cells and p53 wild-type human melanoma UACC-62 cells

**DOI:** 10.1186/1756-0500-3-220

**Published:** 2010-08-03

**Authors:** Xiaoying Zhang, Wei Chen, Ruth Guillermo, Gudiseva Chandrasekher, Radhey S Kaushik, Alan Young, Hesham Fahmy, Chandradhar Dwivedi

**Affiliations:** 1 Department of Biology, Shanghai Chempartner Co., Ltd., Shanghai 201203, China; 2 Department of Pharmaceutical Sciences, South Dakota State University, Brookings, SD 57007, USA; 3 Department of Veterinary and Biomedical Sciences, South Dakota State University, Brookings, SD 57007, USA; 4 Department of Biology and Microbiology, South Dakota State University, Brookings, SD 57007, USA

## Abstract

**Background:**

α-Santalol, an active component of sandalwood oil, has shown chemopreventive effects on skin cancer in different murine models. However, effects of α-santalol on cell cycle have not been studied. Thus, the objective of this study was to investigate effects of α-santalol on cell cycle progression in both p53 mutated human epidermoid carcinoma A431 cells and p53 wild-type human melanoma UACC-62 cells to elucidate the mechanism(s) of action.

**Methods:**

MTT assay was used to determine cell viability in A431 cells and UACC-62; fluorescence-activated cell sorting (FACS) analysis of propidium iodide staining was used for determining cell cycle distribution in A431 cells and UACC-62 cells; immunoblotting was used for determining the expression of various proteins and protein complexes involved in the cell cycle progression; siRNA were used to knockdown of p21 or p53 in A431 and UACC-62 cells and immunofluorescence microscopy was used to investigate microtubules in UACC-62 cells.

**Results:**

α-Santalol at 50-100 μM decreased cell viability from 24 h treatment and α-santalol at 50 μM-75 μM induced G_2_/M phase cell cycle arrest from 6 h treatment in both A431 and UACC-62 cells. α-Santalol altered expressions of cell cycle proteins such as cyclin A, cyclin B1, Cdc2, Cdc25c, p-Cdc25c and Cdk2. All of these proteins are critical for G_2_/M transition. α-Santalol treatment up-regulated the expression of p21 and suppressed expressions of mutated p53 in A431 cells; whereas, α-santalol treatment increased expressions of wild-type p53 in UACC-62 cells. Knockdown of p21 in A431 cells, knockdown of p21 and p53 in UACC-62 cells did not affect cell cycle arrest caused by α-santalol. Furthermore, α-santalol caused depolymerization of microtubules similar to vinblastine in UACC-62 cells.

**Conclusions:**

This study for the first time identifies effects of α-santalol in G_2_/M phase arrest and describes detailed mechanisms of G_2_/M phase arrest by this agent, which might be contributing to its overall cancer preventive efficacy in various mouse skin cancer models.

## Background

Cancer is the second leading cause of death in the United States accounting for one of every four deaths exceeded only by heart diseases. Among all types of cancers, skin cancer including basal cell carcinoma (BCC) and squamous cell carcinoma (SCC) is the most common form of cancer in the United States with more than one million cases diagnosed yearly leading to an estimated 11,590 deaths in 2009 [1]. Therefore, the development of effective chemopreventive or chemotherapeutic agents is useful to address the risk of cutaneous malignancies.

Recently, there has been a considerable interest in the use of naturally occurring agents especially phytochemicals, minerals and vitamins for the chemopreventive activity against various malignancies under both *in vitro *and *in vivo *conditions [2,3]. More than 1000 phytochemicals have shown cancer chemopreventive effects and about 400 of them are currently under further investigation [4-7]. One of such phytochemical is α-santalol which has shown excellent chemopreventive effects against skin cancer under both *in vivo *and *in vitro *conditions [8-12].

α-Santalol is a major component of sandalwood oil (*Santalum album *Linn, Indian sandalwood) which has been traditionally used in the treatment of various skin ailments. Our previous studies reported that topical application of α-santalol (5%, w/v) showed significant chemopreventive effects on 7, 12-dimethylbenz(a)anthracene (DMBA)-initiated and 12-O- tetradecanoylphorbol-13-acetate (TPA)-promoted skin cancer development, and inhibited ornithine decarboxylase (ODC) activity and DNA synthesis induced by TPA in both CD-1 and SENCAR mice [8]; furthermore, α-santalol (5%, w/v) application significantly inhibited skin tumorigenesis by UVB-initiated and TPA-promoted, DMBA-initiated and UVB-promoted, and UVB-initiated and UVB-promoted in SKH-1 hairless mice, and also suppressed UVB-caused induction of epidermal ODC activity in SKH-1 mice [9]. Dose-response experiment indicated that 5% of α-santalol (w/v) application resulted in a relatively higher inhibition of skin tumorigenesis induced by UVB in SKH-1 mice [10]. Both *in vivo *and *in vitro *models suggested that one of the possible mechanisms of its chemopreventive effects is related to induction of apoptosis through both extrinsic and intrinsic pathways [11,12]. In terms of mechanism(s) of chemopreventive agents, in addition to the induction of apoptosis, studies in recent years are focused on the modulation of cell survival pathways such as cell cycle arrest, since disruption of the normal regulation of cell cycle progression and division are important events in the development of cancer [13]. However, to our knowledge, the effects of α-santalol on cell cycle have not been investigated. Thus, the objective of this study was to determine the effects of α-santalol on different phases of cell cycle and proteins required for the regulation of cell cycle progression and arrest in both p53 mutated human epidermoid carcinoma A431 cells and p53 wild-type human melanoma UACC-62 cells.

The results obtained from current study show that in addition to induction of apoptosis by α-santalol as reported previously [12], α-santalol caused G_2_/M phase cell cycle arrest to decrease cell viability. In the mechanistic studies, alterations of cell cycle regulatory proteins and complexes involved in the G_2_/M transition were identified to further elucidate the mechanisms of chemopreventive effects against skin cancer by α-santalol.

## Methods

### Materials and reagents

Propidium iodide, RNase, N-2-hydroxyethylpiperazine-N'-2- ethanesulfonic acid (HEPES), sodium chloride, sodium dodecyl sulfate (SDS), triton, sucrose, phenylmethanesulphonylfluoride (PMSF), anti-α-tublin-FITC antibody were purchased from Sigma Chemical Co. (St. Louis, MO). Dulbecco's modified eagle's medium (DMEM), RPMI-1640 fetal bovine serum (FBS), trypsin EDTA, phosphate buffered saline (PBS) and DPBS were from Mediatech, Inc. (Herndon, VA). Dimethyl sulfoxide (DMSO) was obtained from Fisher Scientific (Fair Lawn, NJ). Leupeptin and pepstatin were from Roche Diagnostics GmbH (Mannheim, Germany). All primary antibodies, secondary antibodies, RIPA lysis buffer, siRNA and siRNA transfection reagent were from Santa Cruz Biotechnology (Santa Cruz, CA). ECL Plus Kit was bought from Amersham Biosciences (Piscataway, NJ). Other reagents were obtained in their highest purity grade available commercially.

### Isolation of α-santalol

α-Santalol was isolated from sandalwood oil (Now Foods, Glendale HTS., IL) by column chromatography using n-Hexane: Ethyl acetate 3:1 as a solvent system. The purity was assessed by gas chromatography [14].

### Cell culture

Human epidermoid carcinoma A431 cell line and human melanoma UACC-62 cell line were purchased from American Type Culture Collection (ATCC, Manassas, VA). A431 Cells and UACC-62 cells were grown in DMEM and in RPMI-1640 medium supplemented with 10% heated inactivated fetal bovine serum, 100 unit/mL of penicillin and 100 μg/mL of streptomycin in a humidified atmosphere containing 5% CO_2 _and 95% air at 37°C.

### α-Santalol solution

α-Santalol was dissolved in DMSO to make 0.05 mol/L stock solution, and stock solution of α-santalol was diluted in growth medium at different concentrations and immediately used. In all the assays, the final concentrations of DMSO in growth medium were 0.4%.

### Determination of cell viability by MTT assay

Cell viability was determined by MTT assay as described by Zhang *et al*. [15]. A431 cells were plated at a density of 7,500 cells/well and UACC-62 cells in a 96-well plate. After 24 hours, cells were treated as vehicle in growth media as control or various concentrations of α-santalol (10-100 μM) for 24, 48, and 72 h. At the end of each treatment, MTT stock solution (5 mg/mL) was diluted to 0.5 mg/mL by medium and immediately used. The medium covering the cells was aspirated off, and then cells were incubated with 50 μL of 0.5 mg/mL MTT solution for 4 hours at 37°C. Thereafter, 150 μL of DMSO was added to each well to dissolve dye crystal formazan and the plate was allowed to stand for 1 hour at 37°C, and then mixed with the microplate shaker for 5 minutes to make sure that all purple crystals were dissolved. Absorbance was measured by SpectraMax M2 microplate reader (Molecular Devices, Sunnyvale, CA) at 570 nm, with the absorbance at 650 nm to correct for background in the presence of an appropriate blank (without cells). The blank reading was subtracted from experimental readings and cell viability was expressed as the percentage of the absorbance values of α-santalol treated groups to untreated controls.

### Analysis of cellular DNA content by flow cytometry

A431 cells or UACC-62 cells were plated in 6-well plates. After 24 hours, cells were treated as either growth medium with 0.4% DMSO alone as control or various concentrations of α-santalol (25 μM, 50 μM and 75 μM) for 6 h, 12 h, 24 h and 48 h respectively. At the end of each treatment periods, cells were harvested by trypsinization and washed twice with ice-cold PBS, and then fixed with ice-cold 70% ethanol in DPBS at 4°C. Fixed cells were then centrifuged and washed with staining buffer. After washing, the pellets were treated with 100 μL RNase A (1 mg/mL) for 30 min at 37°C. After incubation, 900 μL of staining buffer and 20 μL of propidium iodide (1 mg/mL) were added to each sample and incubated in the dark for 30 min. The samples were then analyzed with BD FACScan™ flow cytometry (BD Biosciences, San Jose, CA) using CellQuest Software (BD Biosciences, San Jose, CA).

### Immunoblotting

A431 cells (3.5 × 10^6^) or UACC-62 cells (1 × 10^6^) were plated to each 100 mm culture dish before drug treatment. After 24 hours of cell attachment, cells were treated as either growth medium with 0.4% DMSO alone as control or concentrations of α-santalol (25 μM, 50 μM and 75 μM) for 12 h, 24 h and 48 h respectively. At the end of the treatment, cells were lysed and protein concentrations were determined by BCA™ protein assay kit (Pierce, Rockford, IL) with albumin as a standard as described by Zhang *et al*. [15]. All samples (30-80 μg of proteins) were subjected to 7.5%-15% sodium dodecyl sulfate polyacrylamide gel (SDS-PAGE). The proteins in gels were transferred to nitrocellulose membranes. After blocking the membranes with 5% non-fat milk, membranes were probed with the appropriate dilution of primary antibodies followed by appropriate horseradish peroxidase (HRP) conjugated secondary antibody and ECL Plus detection kit (Amersham Biosciences, Piscataway, NJ) by using a UVP Biochem Gel Documentation system (UVP, Inc., Upland, CA).

### Transfection of small interfering RNA (siRNA)

p21, p53 siRNA and siRNA transfection reagent were purchased from Santa Cruz Biotechnology (Santa Cruz, CA) following Vendor's protocol. Briefly, A431 cells and UACC-62 cell were seeded into 6-well plates for about 24 h, when they had reached about 60% confluence. Then, the cells were incubated with siRNA transfection reagent and siRNA for 10 h. Next, 2 times of normal growth medium was added to the tranfection mixture. After additional 18 h, medium was replaced by normal growth medium for another 18-24 h. Then, the cells were harvested and seeded into 6-well plates and 100 mm dishes. After cell attachment (18-24 h), various concentrations of α-santalol were treated. At end of each treatment (12 h or 24 h), cells were fixed for the analysis of DNA content by flow cytometer and proteins were extracted for the immunoblotting to confirm the efficacy of each transfection. When the transfection efficacy was more than 50%, the data for the analysis of DNA content were used.

### Immunofluorescence microscopy of UACC-62 cells

UACC-62 cells were cultured in chamber slides and treated with different concentrations of α-santalol (50 μM-100 μM), taxol (1 nM-20 nM) and vinblastine (1 nM-20 nM) as two positive controls. After 24 h treatment, cells were fixed with 4% paraformaldehyde. Cells were then blocked with 5% bovine serum albumin and incubated with anti-α-tubulin-FITC and 4'-6-diamidino-2-phenylindole (DAPI) for 2 h at 37°C. Coverslips were mounted and examined with an Olympus AX70 fluorescence microscope.

### Statistical Analysis

Data were analyzed with INSTAT software (Graph Pad, San Diego, CA). ANOVA followed by Tukey post test was applied to compare the statistical difference of different α-santalol treatment groups with untreated controls. Significance in all the experiment was considered at *P *< 0.05. Values were expressed as mean ± the standard deviation of the mean.

## Results

### α-Santalol treatment inhibited cell viability in A431 cells and UACC-62 cells

**Figure 1:**
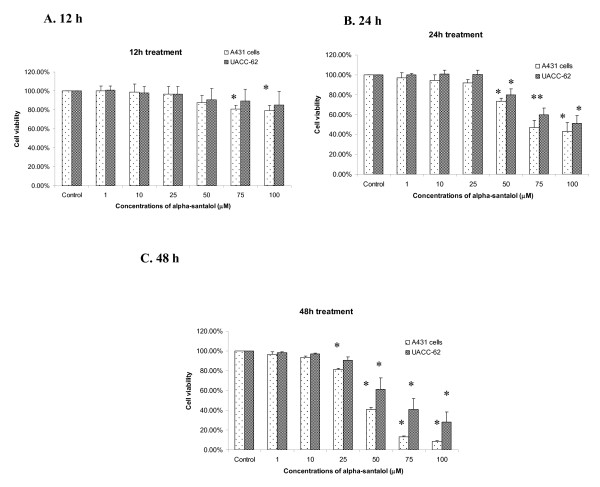
**Effects of α-santalol on cell viability in A431 and UACC-62**. Cells were treated with α-santalol (0-100 μM) for 12 h **(A)**, 24 h **(B) **and 48 h **(C)**. At the end of the respective treatments, MTT assay was performed as detailed in the materials and methods. Values are mean ± SD of three independent observations. *, *P *< 0.05 indicates significantly lower after α-sanatalol treatment as compared to their individual control cells.

Our first aim was to investigate whether α-santalol treatment imparts anti-proliferative effect against human epidermoid carcinoma A431 cells and human melanoma UACC-62 cells, as this is the first study to assess the effect of α-santalol on a solid tumor (UACC-62) cell line. As shown in Figure 1A, for 12 h treatment, α-santalol treatment starting at 75 μM significantly inhibited cell viability of A431 cells as compared to control. For 24 h treatment, α-santalol at 50 μM-100 μM resulted in 26.7%-56.8% and 20.2%-51.1% decrease of cell viability in A431 and UACC-62 respectively as shown in Figure 1B. For 48 h treatment, α-santalol at 50 μM-100 μM inhibited 59.1%-91.6% and 38.9%-71.9% of cell viability as compared to their individual control in A431, and UACC-62 respectively as shown in Figure 1C.

Overall, α-santalol treatment inhibited the cell viability of two cell lines in a concentration- and time-dependent manner. Therefore, further experiments were performed to investigate whether the decrease of cell viability in skin cancer cell lines by α-santalol treatment is related to the alteration in normal cell cycle distribution.

### α-Santalol induced G_2_/M phase cell cycle arrest in A431

In order to better understand the mechanism of inhibition of cell viability, we investigated the effects of α-santalol on various phases of cell cycle in both A431 cells and UACC-62 cells. Analysis of cell cycle phases was performed by flow cytometry after the treatment of cells with α-santalol. To our knowledge, this is the first study to assess the effects of α-santalol on cell cycle progression in any cell line.

**Figure 2:**
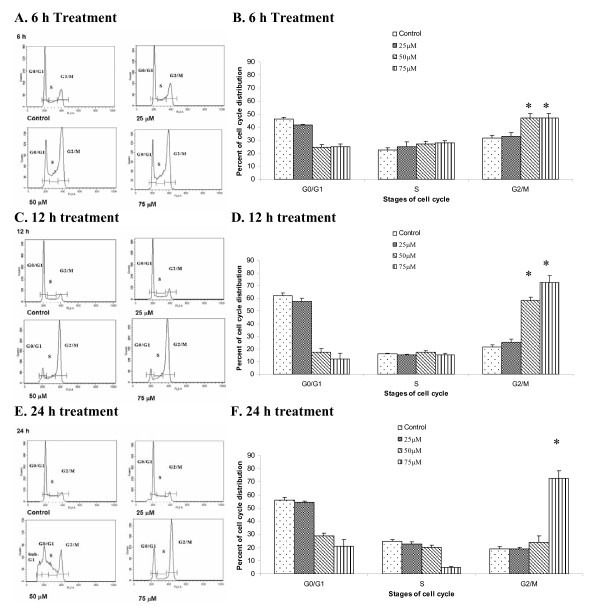
**Effects of α-santalol on the distribution of A431 cells in the different phases of the cell cycle**. A431 Cells were treated with α-santalol (0 μM-75 μM) for 6 h (**A **and **B**), 12 h (**C **and **D**) and 24 h (**E **and **F**). At the end of respective treatment, cells were harvested and digested with RNase. Cellular DNA was stained with propidium iodide and analyzed by flow cytometer as described in the Materials and Methods. Panels **A**, **C **and **E **are histograms representing different time treatment with α-santalol. Data in Panel **B**, **D **and **F **from the cell cycle distribution were summarized and presented as the mean ± SD of three observations. *, *P *< 0.05 indicates statistical significance in α-sanatlol treated groups as compared to the control in A431 cells.

As shown in Figure 2, for A431 cells, α-santalol at 25 μM produced relatively small changes in DNA distribution between all phases of cell cycle. Whereas, starting at 6 h treatment, α-santalol at 50 μM and 75 μM resulted in a significantly (*P *< 0.05) higher number of A431 cells in the G_2_/M phase as measured by the distribution of DNA content compared to the control. Similar findings were observed at 12 h (Figure 2C and 2D) and 75 μM for 24 hr treatment (Figure 2E and 2F). The increase in cell population in G_2_/M phase caused by α-santalol was associated with a corresponding shift in the population of cells mainly in G_0_/G_1 _phase.

However, α-santalol at 50 μM for 24 h treatment in A431 cells did not significantly increase the number of cells in the G_2_/M phase. As shown in Figure 2E, 50 μM of α-santalol for 24 h treatment resulted in a strongly different histogram as compared with other concentrations and other time periods, since a large number of cells (25.2% ± 3.7%) were observed before G_0_/G_1 _phase marked as Sub-G_1_, which were apoptotic cells [16].

### α-Santalol induced G_2_/M phase cell cycle arrest in UACC-62 cells

**Figure 3:**
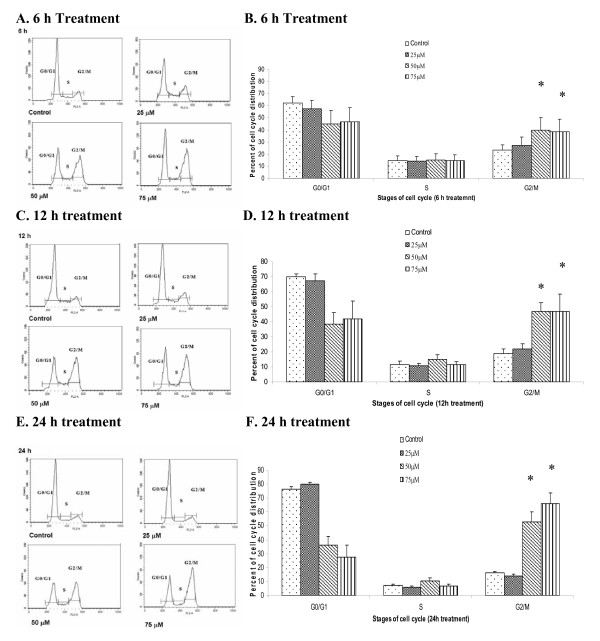
**Effects of α-santalol on the distribution of UACC-62 cells in the different phases of the cell cycle**. UACC-62 Cells were treated with α-santalol (0 μM-75 μM) for 6 h (**A **and **B**), 12 h (**C **and **D**) and 24 h (**E **and **F**). Data were summarized and presented as the mean ± SD of three observations. *, *P *< 0.05 indicates statistical significance in α-sanatlol treated groups as compared to the control in UACC-62 cells.

A431 cells are p53 mutated skin cancer cell line [17]. As p53 plays an important role in regulation of cell cycle progress, the effects of α-santalol on cell cycle progression in a p53 wild-type UACC-62 cell line [18], were also investigated. As shown in Figure 3, for UACC-62 cells, starting at 6 h treatment, α-santalol at 50 μM and 75 μM significantly caused a G_2_/M phase arrest in UACC-62 cells. Similar findings were observed at 12 h (Fig 3C and 3D) and 24 hr treatment (Fig 3E and 3F). The increasing cell population in G_2_/M phase caused by α-santalol is largely due to the decreasing cell population in G_0_/G_1 _phase. Table 1 summarized the percentage of G_2_/M phase induction caused by α-santalol as compared to control in both cell lines. α-Santalol has similar effects on cell cycle progression in UACC-62 cells as well as A431 cells for most concentrations and time periods. These results suggest that α-santalol causes G_2_/M phases arrest in both wild-type and mutant p53 skin cancer cell lines.

**Table 1:** Comparison of G_2_/M phase induction by α-santalol in A431 cells and UACC-62 cells

Time of treatment	Conc. of α-santalol (μM)	Percentage of G_2_/M phase induction by α-santalol as compared to control *	
		
		p53 mutated human epidermoid carcinoma A431 cells	p53 wild-type human melanoma UACC-62 cells
6 h	50	49.1	70.9
	
	75	48.8	65.2

12 h	50	171.1	148.7
	
	75	235.5	149

24 h	50	27.2	226
	
	75	285	305.5

*: Data were expressed as the percentage of the induction of α-santalol treated groups in G2/M phase to untreated controls.

### α-Santalol changed expressions of proteins involved in the G_2_/M phase transition in A431 cells

Cell cycle progression and arrest processes are dependent on the levels of cyclins, cylin-dependent kinases (CDKs) and their inhibitors, thus we have also investigated the changes in the expression of cell cycle protein levels in A431 cells after α-santalol treatment. Marked changes in the expression of proteins that are known to play a role in G_2_/M phase progression were observed in the presence of α-santalol in A431 cells.

**Figure 4:**
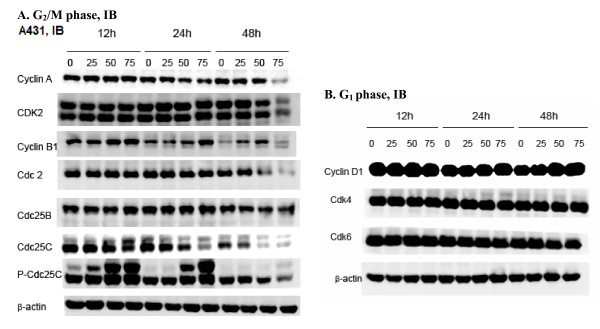
**Effects of α-santalol on cell cycle regulators in A431 cells as determined on the follows, (A) proteins involved in G_2_/M phase and (B) proteins involved in G_1 _phase**. Cells were treated with different concentrations of α-sanatlol for different time periods, and then cells were collected by brief trypsinization. Total cell lysates were prepared and loaded to SDS-PAGE and Western blotting. Membranes were then probed with different primary antibodies followed by appropriate secondary antibody and ECL detection. Photographs of the chemiluminescent detection of the blots, which were representative of three independent experiments, are shown. β-actin was used to verify equal loading of the samples. Data were represented as at least three independent observations. IB, immunoblotting.

As shown in Figure 4A, cyclin A, a protein involved in both S and G2 phase was significantly down-regulated at 48 h time point with 75 μM doses. The expression of Cdk2 which associates with cyclin A for cell cycle progression was found to be decreased at 48 h with 75 μM of α-santalol treatment. Next, cyclin B1, a protein involved in M phase was investigated. Normally, cyclin B degradation occurs at the end of mitotic phase. However, in the presence of α-santalol, the level of cyclin B1 protein was increased as early as 12 h and 24 h treatment at 50-75 μM and remained undegradable till 48 h with 25-50 μM of α-santalol. Cyclin B1 activates Cdc2. Therefore, we determined the impact of α-santalol on the expressions of Cdc2. As presented in the Figure 4A, α-santalol only inhibited the expression of Cdc2 for 48 h treatment and α-santalol did not affect the expression of Cdc2 at other concentrations and other time period treatments.

Cdks are maintained in an inactive state through the phosphorylation of Thr 14 and Tyr 15. The rate limiting step in the activation of Cdks is dephosphorylation of these residues by Cdc25 phosphatases [19]. There are three mammalian cdc25 homologues: Cdc25A, Cdc25B and Cdc25C [19]. In order to better understand the effects of α-santalol on Cdks, we further determined the impact of α-santalol on the expressions of Cdc25A, Cdc25B and Cdc25C in A431 cells. Of all the Cdc25 phosphatases tested, significant changes in the expression pattern were observed only in Cdc25C with α-Santalol. As presented in the Figure 4A, α-santalol caused a concentration- and time-dependent inhibition of Cdc25C expression. Cdc2 can be activated through the dephosphorylation of Thr 14 and Tyr 15 by phospharylating Cdc25C to p-cdc25C at Ser 216 [19]. Even though the expressions of Cdc25C were inhibited by α-santalol treatment, the expressions of p-Cdc25C were increased by α-santalol treatment as one more band of p-Cdc25C was induced as early as 12 h treatment of α-santalol indicating that the up-regulation of p-Cdcd25C by α-santalol treatment might cause A431 cells to decrease the synthesis of Cdc25C

### α-Santalol did not change expressions of proteins involved in the G_1 _phase in A431 cells

Since α-santalol caused cell accumulation in the G_2_/M phase and altered the expression of proteins involving in the G_2_/M phase, we also wanted to know whether α-santalol had effects on proteins involving in the G_1 _phase in A431 cells. As shown Figure 4B, various concentrations of α-santalol for different time period treatments did not alter the expressions of cyclin D1, Cdk4 and Cdk6. These results indicate that α-santalol specifically influences G_2_/M phase cell cycle progression in A431 cells.

### α-Santalol inhibited expressions of proteins involved in the G_2_/M phase transition in UACC-62 cells

**Figure 5:**
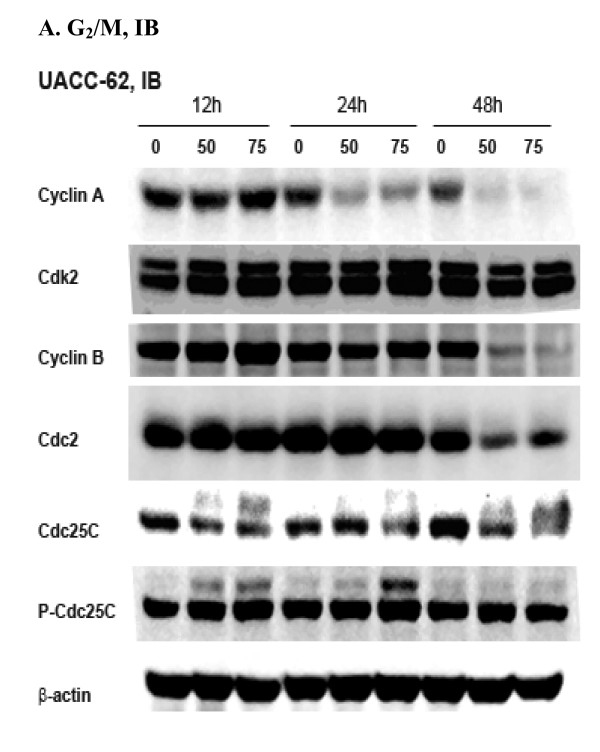
**Effects of α-santalol on cell cycle regulators in UACC-62 cells as determined on proteins involved in G_2_/M phase**. Data were represented as at least three independent observations.

Since α-santalol caused G_2_/M phase arrest in both A431 cells and UACC-62 cells, we wanted to know whether molecular events that are responsible for G_2_/M phase arrest caused by α-santalol treatment would be same in both cell lines. Thus, expressions of G_2_/M phase regulatory proteins such as cyclin A, Cdk2, cyclin B, Cdc2, Cdc25c and p-Cdc25C were assessed in UACC-62 cells as well. As shown in Figure 5A, α-santalol at 50 μM and 75 μM almost completely blocked the expressions of cyclin A for 24 h and 48 h treatment in UACC-62 cells. The expressions of cyclin B were unchanged at 12 h and 24 h treatment, but significantly inhibited at 48 h treatment in UACC-62. p-Cdc25C remained unchanged with α-santalol treatment. The effects of α-santalol on other proteins such as Cdk2, Cdc2, Cdc25C were similar in A431 cells and UACC-62 cells.

### α-Santalol induced expression of p21 and inhibited expression of mutant p53 in A431 cells

**Figure 6:**
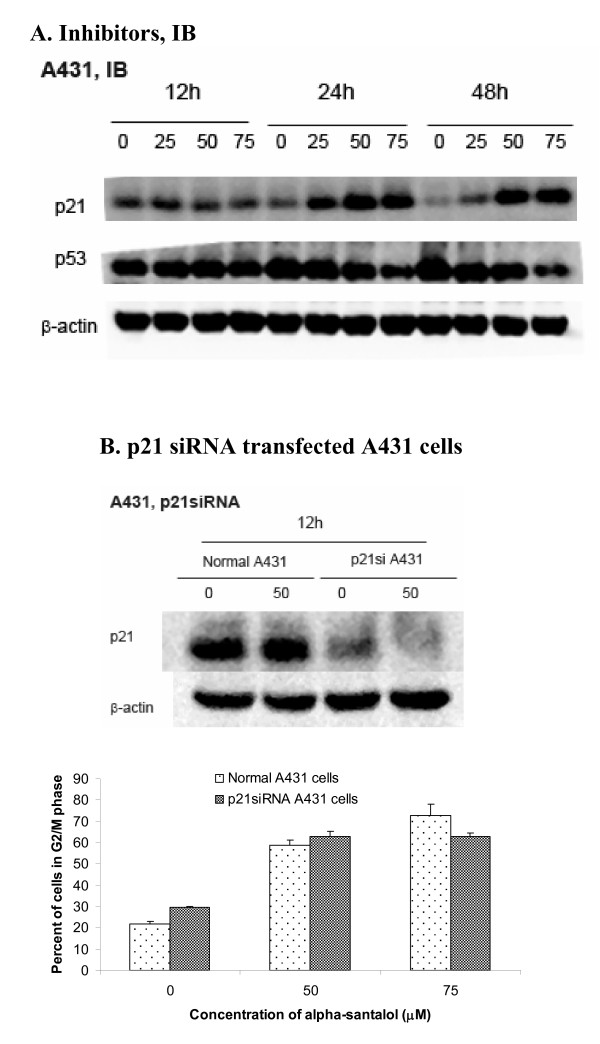
**Effects of α-santalol on p21 and p53 in A431 cells**. **(A)**. Protein levels of p21 and p53 after α-santalol treatment in A431 cells were evaluated by Western blotting. **(B)**. Cell cycle distribution in p21 siRNA transfected A431 cells was analyzed by flow cytometer. Data were summarized and presented as the mean ± SD of three observations. For each p21 siRNA transfection in A431 cells, Western blotting was performed to ensure the inhibition of p21 protein expression.

In addition to cyclins and CDKs, p21 is a cyclin-dependent kinase (cdk) inhibitor, which tightly regulates the activities of cyclin/CDK enzyme complex. As shown in Figure 6A, in A431 cells, the expression of p21 was strongly increased starting at 25 μM of α-santalol for 24 h treatment. p21 can be activated through either p53-dependent pathway or p53-indepedent pathway. Since A431 cells are p53 mutated cell line (19), the up-regulation of p21 by α-santalol in A431 cells is not mediated through a p53-dependent pathway. Moreover, it was found that expressions of mutated p53 were decreased after treatment of different concentrations of α-santalol as shown in Figure 6A. Inhibition of mutated p53 may also contribute to the chemopreventive effects of α-santalol *in vivo *animal models [8-11].

### G_2_/M phase arrest caused by α-santalol is not dependent on p21 in A431 cell

In order to elucidate the role of upregulation of p21 on its overall cell cycle progression by α-santalol, p21 siRNA was used to inhibit p21 expression in A431 cells as confirmed by Western blotting. As shown in Figure 6B, the percentage of cells in G_2_/M phase caused by α-santalol treatment was similar in both normal A431cells and p21 down-regulated A431 cells, which suggested that G_2_/M phase arrest caused by α-santalol is not dependent on p21 in A431 cells.

### α-Santalol induced expression of p21 and p53 in UACC-62 cells

**Figure 7:**
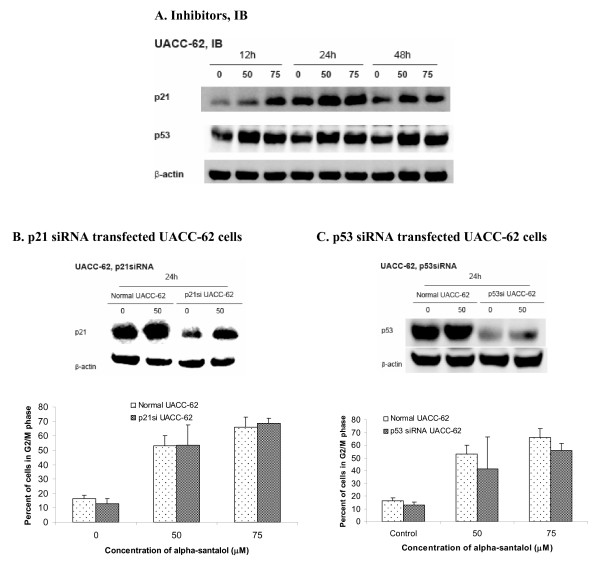
**Effects of α-santalol on p21 and p53 in UACC-62 cells**. **(A)**. Protein levels of p21 and p53 after α-santalol treatment in UACC-62 cells were evaluated by Western blotting. Cell cycle distribution in **(B) **p21 siRNA and **(C) **p53 siRNA transfected UACC-62 cells was analyzed by flow cytometer. Data were summarized and presented as the mean ± SD of three observations. For each p21 siRNA or p53 siRNA transfection in UACC-62 cells, Western blotting was performed to ensure the inhibition of p21 or p53 protein expressions.

In UACC-62 cells, the expressions of both p21 and wild-type p53 were increased by α-santalol treatment starting from 12 h treatment as shown in Figure 7A. However, as can be seen from Figure 7B and 7C, either down-regulation of p21 or p53 in UACC-62 cells by siRNA did not significantly changed percentage of cells in G_2_/M phase caused by α-santalol as compared to normal UACC-62 cells. These results revealed that G_2_/M phase arrest caused by α-santalol in UACC-62 cells is not dependent on both p21 and p53.

### α-Santalol caused disruption of the cellular microtubule network in UACC-62 cells

**Figure 8:**
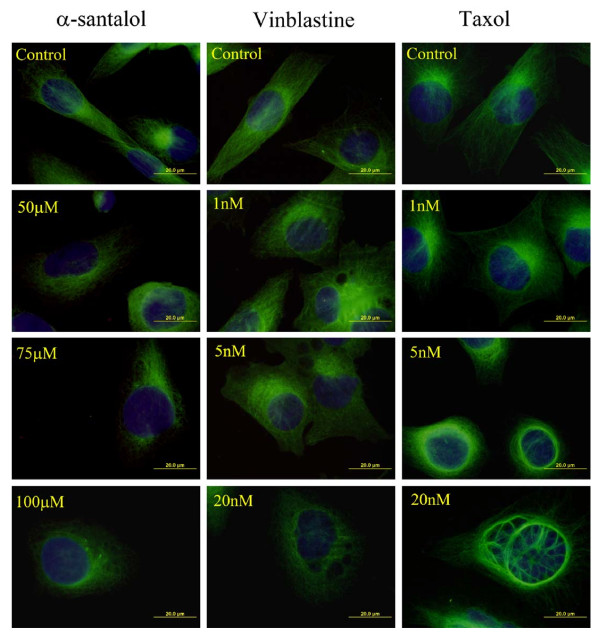
**Effects of α-santalol (50 μM-100 μM), taxol (1 nM-20 nM) and vinblastine (1 nM-20 nM) on microtubules in UACC-62 cells**. Cells were exposed to drug for 24 h before fixation and staining with an anti-tubulin antibody as described in Materials and Methods. Microtubule morphology (green) and nuclei conterstained with DAPI (blue) were examined by immunofluorescence microscopy.

Most antimicrotubule agents that target the cellular microtubule network result in an aberrant formation of the mitotic spindle, subsequent blockage of the cell cycle in G_2_/M phase and causing apoptotic cell death [20,21]. Since α-santalol caused G_2_/M phase arrest and apoptosis, we examined whether it affects the organization of microtubules in UACC-62 cells, which are relatively big cells and their microtubules are easily visualized compared to A431 cells. UACC-62 cells were treated with different concentrations of α-santalol (50 μM-100 μM), taxol (1 nM-20 nM), vinblastine (1 nM-20 nM) as presented in Figure 8. After 24 h treatment, microtubules in the control cells were clearly seen to traverse intricately throughout the cell and individual microtubules often appeared long and relatively straight. In contrast, α-santalol caused a dose-dependent loss of microtubule network, since the antibody-based fluorescence of microtubules was dispersed throughout the cytoplasm. These effects are similar to that exerted by vinblastine, a known inhibitor of tubulin polymerization, but different from that of taxol which stabilized microtubules causing them to form long polymerized microtubule bundles. Thus, we concluded that α-santalol likely caused microtubule depolymerization in UACC-62 cells.

## Discussion

In our previous studies, α-santalol, a naturally occurring terpenoid, has shown to have chemopreventive effects on both DMBA-initiated and TPA-induced skin cancer development in CD-1 and SENCAR mice [8] and UVB-induced skin tumor developments in SKH-1 hairless mice [9-11]. *In vitro *studies also demonstrated that α-santalol could be effective against skin carcinogenesis in mouse models through induction of apoptosis via caspase activation together with dissipation of mitochondria membrane potential and cytochrome *c *release in A431 cells [12].

The development of cancer is also associated with disorders in the regulation of the cell cycle in addition to loss of apoptosis [13,22]. During the cell cycle, mammalian cells coordinate cell growth, genome replication and cell division. Two irreversible events subdivided the cell cycle into distinct phases: DNA replication of cells is defined as S phase and cell division is called as M phase. G_1 _and G_2 _are two gap phases where cells grow and carry out additional functions [23].

Even though our previous studies showed that α-santalol could induce apoptosis through caspase-dependent pathway in both *in vivo *[11] and *in vitro *in A431 cells [12], the effects of α-santalol on the cell cycle have not been studied yet. Accordingly, in this study, the effects of α-santalol on cell cycle and proteins involved in the cell cycle regulation in two human skin cancer-derived cancer cell lines, epidermoid carcinoma A431 cells and melanoma UACC-62 cells were investigated. The hypothesis in this study was whether α-santalol could affect the cell cycle progression which may contribute to its chemopreventive effects in animal models.

Our data demonstrated that treatment of α-santalol resulted in a concentration- and time- dependent inhibition of cell viability on A431 cells and UACC-62 cells as determined by MTT assay. Treatment of α-santalol (50 μM) for 12 h did not significantly decrease cell viability of A431 and UACC-62 cells. However, flow cytometric analysis of cell cycle distribution revealed that α-santalol (50-75 μM) from 6 h treatment to 24 h treatment led to a 49%-285% and 71%-306% induction of G_2_/M phase in A431 cells and UACC-62 cells respectively as compared to control cells. These findings indicated that G_2_/M phase cell cycle arrest induced by α-santalol treatment may be one of the mechanisms which results in a decrease of cell viability in A431 and UACC-62 cells after α-santalol treatment.

Cell cycle progression is tightly regulated by cyclin/cyclin-dependent kinase (Cdks) complexes [24-27]. For instance, cyclin D/Cdk4 and Cdk6 drive the sequential progression from G_1 _to S phase; cyclin A/Cdk2 and Cdc2 (Cdk1) complexes control the S and G_2 _phases; and cyclin B/Cdc2 complex drives the G_2_/M transition as well as processes during mitosis [23]. Accordingly, in the present paper, the effects of α-santalol on proteins involved in the G_2_/M phase were first investigated. Results revealed that α-santalol treatment has different impacts on cell cycle regulatory proteins in A431 cells and UACC-62 cells, even through α-santalol caused G_2_/M phase arrest in both cell lines.

In A431 cells, expressions of cyclin A, Cdk2 and Cdc2 were decreased only after treated by a high concentration of α-santalol (75 μM) for 48 h. However, expressions of cyclin B1 were increased as early as 12 h treatment. In UACC-62 cells, expressions of cyclin A were fully inhibited starting from 24 h treatment and both cyclin A/Cdk2 and cyclin B1/Cdc2 complexes were decreased by α-santalol treatment. It is known that the activation of cyclin B1/Cdc2 complex is required to entry into mitosis through dephosphorylation of inhibitory sites of Cdc2 by phosphorylating Cdc25C to p-Cdc25C [28]. As we compared immunoblotting data of p-Cdc25C in A431 cells and UACC-62 cells, it was found that one more band of p-Cdc25C was induced by α-santalol treatment in A431 cells only which may contribute to the activation of cyclin B/Cdc2 complex in A431 cells. Moreover, cyclin B degradation is essential for completion of mitosis; whereas overexpression of stable cyclin B1 entailed metaphase arrest [29]. Thus, increasing cyclin B1 by α-santalol treatment in A431 cells might induce cell cycle arrest in the mid-metaphase of mitosis [30]; whereas, decreasing cyclin A and cyclin B1 by α-santalol in UACC-62 cells might induce cell cycle arrest in G_2 _phase before entry into mitosis [31].

Since A431 cells express the mutated form of the P53 gene with substitution at codon 273 (His273) [17] and UACC-62 cells are wild-type p53 cells [18], we wanted to know whether p53 plays any role on the observed differences between two cell lines. P53, a tumor suppressor acts as a checkpoint regulator of cell cycles, contributing to cell cycle arrest in the G_1 _[32,33], G_2 _[34,35] phases by a multiple pathways. In the classical p53-dependent pathway G_2_/M phase arrest, activation of p53 interacts with response elements present on the promoter region of p21 to increase expression of p21, which subsequently interacts with CDKs and cyclins to affect cell cycle arrest [23]. Here in the present studies, the expressions of p21 were increased by α-santalol treatment in both cell lines; the expressions of mutated p53, that is overexpressed in most of cancer cells [36], were decreased after α-santalol treatment in A431 cells and expressions of wild-type p53 were increased by α-santalol in UACC-62 cells, all of which may contribute to α-santalol's overall chemopreventive effects against skin cancer. However, knockdown of either p21 or wild-type p53 did not change G_2_/M phase arrest caused by α-santalol, which suggested that α-santalol induced G_2_/M phase arrest independently of p21 and p53.

## Conclusion

The present study demonstrated that in addition to induction of apoptosis by α-santalol observed in *in vitro *and *in viv*o studies [11,12], α-santalol also inhibits human epidermoid carcinoma A431 cell growth and human melanoma UACC-62 cells *in vitro *through G_2_/M phase arrest independently on p21 and p53. α-Santalol may cause metaphase of mitosis arrest in A431 cells through up-regulation of cyclin B; whereas, in UACC-62 cells, α-santalol induced cell arrest in G_2 _phase by down-regulation of both cyclin A and cyclin B complexes resulting in microtubule depolymerization. To our knowledge, this study for first time to report the effects of α-santalol on melanoma cells, which may provide data for the future study of α-santalol on melanoma cancer in animal models. Based on this study, α-santalol could be a potential agent against skin cancer development. Future studies on the effects of α-santalol on detailed mechanisms of cell cycle arrest and other signal pathways for *in vitro *cell lines and *in vivo *animal models are needed to further elucidate the detailed mechanism(s) of action of α-santalol on skin cancer chemoprevention.

## Competing interests

The authors declare that they have no competing interests.

## Authors' contributions

XZ carried out the overall studies and wrote the manuscript. WC helped with the cell cycle analysis, immunoblotting and immunofluorescence microscopy. RG carried out extraction of α-santalol. GC provided valuable suggestion on the experiment on cell cycle part. RSK and AY helped in the flow cytometeric analysis of cell cycle distribution. HF designed the protocol for extraction of α-santalol. CD conceived the study, and participated in its design and preparation of the manuscript. All authors have read and approved the final manuscript.
